# ZiBuPiYin Recipe Prevented and Treated Cognitive Decline in ZDF Rats With Diabetes-Associated Cognitive Decline *via* Microbiota–Gut–Brain Axis Dialogue

**DOI:** 10.3389/fcell.2021.651517

**Published:** 2021-08-18

**Authors:** Tingting Bi, Ruiqi Feng, Libin Zhan, Weiming Ren, Xiaoguang Lu

**Affiliations:** ^1^School of Traditional Chinese Medicine and School of Integrated Chinese and Western Medicine, Nanjing University of Chinese Medicine, Nanjing, China; ^2^Jiangsu Collaborative Innovation Center of Chinese Medicinal Resources Industrialization, Nanjing University of Chinese Medicine, Nanjing, China; ^3^Department of Emergency Medicine, Zhongshan Hospital, Dalian University, Dalian, China

**Keywords:** diabetes-associated cognitive decline, microbiomics and metabolomics, microbiota–gut–brain axis, ZiBuPiYin recipe, prevention

## Abstract

Gut microbiota is becoming one of the key determinants in human health and disease. Shifts in gut microbiota composition affect cognitive function and provide new insights for the prevention and treatment of neurological diseases. Diabetes-associated cognitive decline (DACD) is one of the central nervous system complications of type 2 diabetes mellitus (T2DM). ZiBuPiYin recipe (ZBPYR), a traditional Chinese medicine (TCM) formula, has long been used for the treatment of T2DM and prevention of DACD. However, the contribution of ZBPYR treatment to the interaction between the gut microbiota and metabolism for preventing and treating DACD remains to be clarified. Here, we investigate whether the gut microbiota plays a key role in ZBPYR-mediated prevention of DACD and treatment of T2DM *via* incorporating microbiomics and metabolomics, and investigate the links between the microbiota–gut–brain axis interaction and the efficacy of ZBPYR in ZDF rats. In the current study, we found that ZBPYR treatment produced lasting changes in gut microbiota community and metabolites and remotely affected hippocampus metabolic changes, thereby improving memory deficits and reversing β-amyloid deposition and insulin resistance in the brain of ZDF rats from T2DM to DACD. This may be related to a series of metabolic changes affected by gut microbiota, including alanine, aspartic acid, and glutamic acid metabolism; branched-chain amino acid metabolism; short-chain fatty acid metabolism; and linoleic acid/unsaturated fatty acid metabolism. In summary, this study demonstrates that prevention and treatment of DACD by ZBPYR partly depends on the gut microbiota, and the regulatory effects of bacteria-derived metabolites and microbiota–gut–brain axis are important protective mechanisms of ZBPYR.

## Introduction

The prevalence of type 2 diabetes mellitus (T2DM) and its complications have increased year by year in the elapsed decades (Groeneveld et al., 2018; Hughes et al., 2018; Xue et al., 2019; Ma et al., 2020). Diabetes-associated cognitive decline (DACD) is a common central nervous system (CNS) complication of T2DM, and its clinical manifestations are mostly learning and memory loss, inattention, and cognitive deficits. It is the most common cause of dementia in the elderly (Fischer et al., 2009; Samaras et al., 2014; Koekkoek et al., 2015; Venkat et al., 2019; Wu et al., 2020). It is now commonly known that β-amyloid (Aβ) deposition and insulin resistance in the brain are the unfavorable outcomes of DACD (Yagihashi et al., 2011; Spauwen and Stehouwer, 2014; Wang and Jia, 2014; Bae et al., 2019). Continuous high blood glucose can induce structural and functional abnormalities in the brain and aggravate cognitive impairment, resulting in DACD (Yagihashi et al., 2011; Palta et al., 2017). Despite the fact that intensive efforts have been made in the diagnosis and therapy of DACD, no definitive therapeutic methods are available for treating DACD. Therefore, discovering the novel mechanisms of DACD and identifying potential therapeutic targets are essential for preventing and treating the development of DACD from T2DM.

Emerging evidence has indicated that the gut microbiota may play a key role in mediating the pathogenesis and progression of T2DM to DACD (Yu et al., 2019; Liu et al., 2020). Generally, there is a beneficial symbiotic relationship between the commensal gut microbiota and the host. Multiple bacteria-derived metabolites have been identified, giving rise to mediate mutualism between the host brain and the intestine (Frohlich et al., 2016). This intimate connection defines the term microbiota–gut–brain axis (Wang and Kasper, 2014; Cryan et al., 2019). It has been found that the microbiota–gut–brain axis plays important roles in several neurological disorders, and targeting the microbiota–gut–brain axis may help ameliorate cognitive impairment (Chen D. et al., 2017). Moreover, the gut microbiota of patients with cognitive impairment differs taxonomically from that of controls, which are manifested as reduced diversity with a distinct taxonomic composition, by increasing the abundance of *Firmicutes* and decreasing the abundance of *Bacteroidetes* at the phylum level, and differences in the abundance of bacteria at the genus level (Chen et al., 2019; Kang et al., 2020). Cognitive pathology can be restored by transferring the healthy microbiota, and cognitive dysfunction can be improved by restoring gut microbiota (Hazan, 2020). Gut microbiota promotes the energy metabolism, immunity, and brain health of the host *via* microbiota metabolites, such as amino acids and short-chain fatty acids (SCFAs) (Heianza et al., 2019; Li et al., 2019). These results demonstrated that disruptions in gut microbiota may play an important role in the development and progression of DACD, and a deeper investigation of DACD-related microbiota structure may provide novel insight into the early diagnosis and development of treatment strategies for the microbiota–gut–brain axis during the development of DACD from T2DM.

Traditional Chinese medicine (TCM) has a history of more than 2,000 years and has been widely used in clinical applications in metabolic and neurological diseases. In recent years, gut microbiota has emerged as a novel and important field for understanding TCM (Feng et al., 2019). Compelling evidence supports the hypothesis that the interactions between TCM and gut microbiota could lead to changes in microbiota and metabolic components (He W. J. et al., 2020). ZiBuPiYin recipe (ZBPYR), an ancient TCM formula recorded in the book of Bujuji written by Wu Cheng in the Qing dynasty, is derived from Zicheng Decoction and has been widely used for the treatment of T2DM and DACD in clinical practice (Sun et al., 2016; Chen J. et al., 2017; Bi et al., 2020). Its chemical characterization, quality control standard, and pharmacokinetics have been reported in our previous studies (Zhu et al., 2014; Dong et al., 2016). Therefore, further investigation of ZBPYR on the prevention and treatment of DACD *via* gut microbiota-mediated insight may help us understand its mechanism of action and valuable clinical applications.

Here, we characterized the phenotype of microbiome and metabolome in ZDF rats and identified the key biomarkers and metabolic structures of ZBPYR against DACD by using a comprehensive strategy combining 16S rRNA genetic sequencing and ultra-performance liquid chromatography coupled to tandem mass spectrometry (UPLC-MS/MS) techniques. It is worth noting that the ZDF rat is a leptin receptor-deficient model, which develops into a stable T2DM model at 9 weeks of age (Zhou et al., 2019) and has symptoms of cognitive dysfunction at 15 weeks of age (Bi et al., 2020). Leptin is involved in the development of the rat embryonic brain (Udagawa et al., 2006), and the lack of leptin receptors may not be the main cause of cognitive decline (Dinel et al., 2011). We aimed to interpret the mechanism of action of ZBPYR as therapy for DACD and to provide the theoretical basis for the clinical evaluation and diagnosis of DACD.

## Materials and Methods

### Animals

Male Zucker diabetic fatty (ZDF, *fa/fa*) rats and lean control Lean Zucker (LZ, *fa*/+) rats, all at 5 weeks old, were purchased from Vital River Laboratories (VRL) (Beijing, China). After arrival, the rats were acclimatized for 3 days under a standard specific-pathogen-free (SPF) environment (23°C, 12 h/12 h light/dark, 65% humidity, and *ad libitum* access to food and water) prior to experiment. The animal experiment protocol was designed to minimize the pain or discomfort of animals and has been approved by the Animal Ethics Committee of Nanjing University of Chinese Medicine (permit number: 201901A009).

### Preparation and Administration of ZBPYR

All medicinal materials of ZBPYR were provided by the Sanyue Chinese Traditional Medicine Co., Ltd. (Nantong, China), as shown in Table 1. The certificate specimens of each herb were identified by the company, and quality inspection reports were provided. All medicinal materials were accurately weighed and boiled twice in distilled water at eight volumes per weight (1:8, w/v) for 2 h. The aqueous solution was passed through eight layers of medical gauze, and the filtered solutions were condensed *in vacuo* and subsequently freeze-dried to provide ZBPYR powder for experimental use. The lyophilized ZBPYR powder was thoroughly dissolved in distilled water for animal administration.

**TABLE 1:** The ingredients of ZBPYR.

Herbal name	Species name and Latin name	Place of origin	Part used	Voucher specimens	Amount used (g)
Hong-Shen	*Panax ginseng* C. A. Mey.	Jilin	Root	180109	30
Shan-Yao	*Dioscorea polystachya* Turcz.	Henan	Rhizome	171207	15
Fu-Shen	*Poria cocos* (Schw.) Wolf	Anhui	Root	171130	15
Bai-Shao	*Paeonia lactiflora* Pall.	Anhui	Root	171123	15
Dan-Shen	*Salvia miltiorrhiza* Bunge	Shandong	Root	171122	12
Bai-Bian-Dou	*Lablab purpureus* (L.) Sweet	Zhejiang	Bean	180111	15
Lian-Zi	*Nelumbo nucifera* Gaertn.	Hunan	Seed	171118	20
Shi-Chang-Pu	*Acorus gramineus* Sol. ex Aiton	Zhejiang	Rhizome	171209	10
Yuan-Zhi	*Polygala tenuifolia* Willd.	Hebei	Root	171013	10
Tan-Xiang	*Santalum album* L.	Guangdong	Sandalwood	180109	4.5
Ju-Hong	*Citrus maxima* “Tomentosa”	Sichuan	Epicarp	171016	9
Gan-Cao	*Glycyrrhiza uralensis* Fisch. ex DC.	Neimenggu	Root	170815	9
Total amount					164.5

### Chemical Composition of ZBPYR

Ultraperformance liquid chromatography–electrospray ionization–quadrupole–time of flight–mass spectrometry (UHPLC-ESI-Q-TOF-MS) was performed on the Shimadzu LC10ATVP high-performance liquid chromatograph (Shimadzu Corporation, Japan) combined with the AB Sciex TripleTOF 5600+ Time-of-Flight Mass Spectrometer (AB SCIEX, Foster City, CA, United States). The separation was performed on an ACQUITY UPLC BEH C18 column (100 mm × 2.1 mm, 1.7 μm), the column temperature was 40°C, and injection volume was 2 μl. The mobile phase was 0.1% formic acid aqueous solution (A)–acetonitrile (B), gradient elution: 0→15 min, 5% B→50% B; 15→20 min, 50% B→80% B; 20→25 min, 80% B→80% B; the flow rate was 0.4 ml/min. The MS conditions were set to positive and negative ion mode; CUR: 35,000 psi; GS1: 55,000 psi; GS2: 55,000 psi; TEM: 550,000°C; CE: −10,000 V (negative ion mode); DP: −60,000 V (negative ion mode); scan range m/z 100–1000 and 50–1000.

### Experimental Design

ZDF rats were randomly divided into eight groups based on body weight and random blood glucose (*n* = 10 per group): 9w-Z group (ZDF model rats treated with distilled water for 4 weeks), 9w-HZ group (ZDF model treated with high-dose ZBPYR for 4 weeks), 9w-MZ group (ZDF model treated with medium-dose ZBPYR for 4 weeks), 9w-LZ group (ZDF model treated with low-dose ZBPYR for 4 weeks), 15w-Z group (ZDF model rats treated with distilled water for 10 weeks), 15w-HZ group (ZDF model treated with high-dose ZBPYR for 10 weeks), 15w-MZ group (ZDF model treated with medium-dose ZBPYR for 10 weeks), and 15w-LZ group (ZDF model treated with low-dose ZBPYR for 10 weeks). LZ rats were randomly divided into two groups (*n* = 10 per group): 9w-L group (LZ control rats treated with distilled water for 4 weeks) and 15w-L group (LZ control rats treated with distilled water for 10 weeks) as controls. During the 4-week (9w group) or 10-week (15w group) experiment, ZBPYR was administered by oral gavage daily at a dosage of 34.6 g/kg, 17.3 g/kg, and 8.7 g/kg to the high-, medium-, and low-dose ZBPYR treatment groups, respectively. These dosages were calculated from the equivalent conversion of the body surface area between animals and humans. The control and model groups were orally administered with the same amount of distilled water instead of ZBPYR. After 4 weeks and 10 weeks of the experiment, levels of random blood glucose (RBG), glycosylated hemoglobin (HbA1c%), fasting serum insulin (FSI), body weight (BW), and abdominal circumference (AC) were measured. Oral glucose tolerance test (OGTT) and insulin secretion test (ITT) were performed, and the homeostasis model of assessment for insulin resistance (HOMA-IR) index was calculated.

### Morris Water Maze (MWM)

The spatial memory ability of 15-week-old rats was performed by the MWM test as described in our previous study (Bi et al., 2020). All rats were trained in a round water pool at 22 ± 2°C with four equal quadrants. Four training trials were performed each day for four consecutive days in the orientation navigation test. In each trial, the rat was given 120 s to find the hidden escape platform. Escape latency was recorded when the rat reached the platform. If the rat did not reach the platform within 120 s, the time was recorded as 120 s. The platform was removed after the orientation navigation test was completed on the fourth day, and the spatial exploration test was carried out by allowing the rat to swim for 120 s. Topscan software was connected to an overhead camera to record and automatically analyze the swimming path of the rat.

### Sample Collection

The rats were euthanized with isoflurane and decapitated for sample collection. All samples of rat intestinal contents were collected, quick-frozen in liquid nitrogen, and stored at −80°C for further procedures. Blood samples were centrifuged (3,000 rpm, 15 min) for serum separation, and all serum aliquots were stored at −80°C. Rat brain tissues (*n* = 3 per group) were fixed for histological staining. Hippocampus and cortex were separated from the remaining rat brain tissues (*n* = 7 per group) and kept at −80°C for further procedures.

### Enzyme-Linked Immunosorbent Assay (ELISA) Analysis

The rat hippocampus and cortex tissues were lysed in lysis buffer supplemented with protease inhibitors and centrifuged according to the manufacturer’s instructions. The concentrations of Aβ were quantified with ELISA kits (Wako, Japan). The absorbance was read at 450 nm using a micro-plate reader and then calculated as a concentration using a standard curve.

### Western Blotting

Hippocampus and cortex tissues were prepared using RIPA lysis buffer (Beyotime, China) containing 1% protease and phosphatase inhibitor cocktail (Cell Signaling Technology, United States). Total protein concentration was determined using a Minim Spectrophotometer, and 20 μg of protein was separated with 8% SDS-PAGE. Separated proteins were transferred onto PVDF membranes (Millipore, United States). After blocking with 5% fat-free milk in TBST for 2 h, the membranes were incubated with the following antibodies at 4°C overnight: phospho-Insulin Receptor Substrate 2 (IRS2) (Ser371) (ab3690, Abcam, 1:1000), IRS2 (4502S, CST, 1:1000), phospho-Protein Kinase B (AKT) (Ser473) (4060s, CST, 1:1000), AKT (9272s, CST, 1:1000), Forkhead Transcription Factor 1 (FOXO1) (2880S, CST, 1:1000), and β-actin (3700S, CST, 1:1000). Membranes were then incubated with secondary antibodies for 90 min. Finally, membranes were chemically exposed and semiquantitatively analyzed with ImageQuant TL 1D system (GE Healthcare, United States). All experiments were performed in triplicate.

### Congo Red Staining

The whole brain of each rat was fixed in 4% paraformaldehyde (Solarbio, China) for 24 h and dehydrated by passaging through a graded series of sucrose solutions. The brain then was embedded in ice-chilled OCT gel, and coronal sections from the hippocampus and cortex were prepared at 20 μm thick. For labeling compact amyloid plaques, brain slices were stained with 1% Congo red solution (Sigma-Aldrich, United States) in 80% absolute ethanol and 1% NaOH. After being washed, the brain sections were counterstained with cresyl violet, dehydrated in absolute ethanol, and then clarified in xylene. The slices were evaluated under a light microscope (OLYMPUS, Japan) at a magnification of 40×.

### Microbiota Community Analysis

The QIAamp Rapid DNA Mini Kit (Qiagen, United States) was used to extract total DNA from the intestinal contents samples according to the manufacturer’s procedures. After determining the DNA concentration and integrity, an amplicon sequencing library was constructed based on the PCR-amplified V3–V4 variable region of 16S rRNA. Then, use qualified libraries on the Illumina MiSeq platform for paired-end sequencing according to the manufacturer’s instructions. Use Trimmomatic, FLASH, and QIIME software to filter the original sequencing data. Then, UPARSE software with a 97% threshold was used to cluster the clean readings into operational taxonomic units (OTUs). Use the QIIME package to select the representative read from each OTU. Use the ribosome database item classifier v.2.2 to annotate and classify representative OTU sequences. The datasets generated in this study have been deposited in the NCBI Sequence Read Archive (SRA) database (Accession Number: SRP299194).

### Metabolomics Profiling Analysis

Metabolomics profiling analysis was performed with intestinal contents and hippocampus tissues as previously described (Zhang L. et al., 2020). The samples were homogenized and centrifuged, and the supernatants were combined. Use the robotic multifunctional MPS2 (Gerstel, Germany) to automatically derive and separate samples. The UPLC-MS/MS system (ACQUITY UPLC-Xevo TQ-S, United States) was used to quantify the microbial metabolites. The reserved solutions of all 132 representative reference chemicals of microbial metabolites were prepared in methanol, ultrapure water, or sodium hydroxide solution. Add internal standards to monitor data quality and compensate for matrix effects. The original data generated were processed with proprietary software XploreMET (v2.0, Metabo-Profile, Shanghai, China) to automatically remove baseline values, smooth and pick peak values, and align peak signals. Principal component analysis (PCA) and supervised partial least squares discrimination analysis (PLS-DA) were performed to visualize metabolic differences among the experimental groups. Differential metabolites were selected according to the orthogonal partial least squares discriminant analysis (OPLS-DA) model, including VIP > 1 and *p* < 0.05. The MetaboAnalyst online tool (version 4.0) was used to explore biological patterns, functions, and pathways of identified differentially expressed metabolites.

### Statistical Analysis

All data were expressed as the mean ± standard deviation (SD), and statistical analyses were performed using GraphPad Prism 8.0 software (GraphPad Software, United States). Significance differences among groups with univariate analysis were determined by one-way ANOVA followed by Tukey’s *post hoc* test. Student’s *t*-test was used to compare two groups. *p* < 0.05 was considered significant.

## Results

### The Chemical Composition of ZBPYR

**FIGURE 1:**
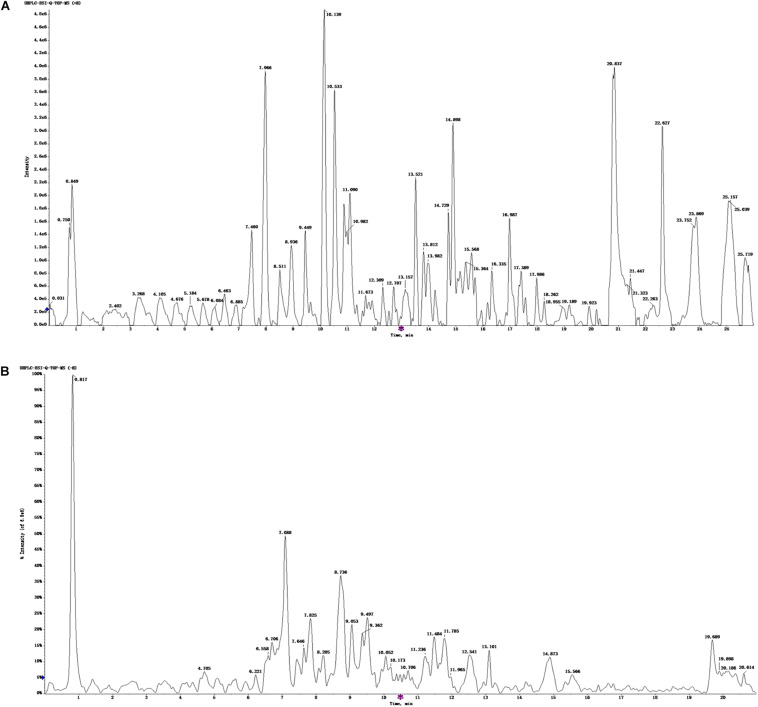
UHPLC-ESI-Q-TOF-MS total ion chromatogram of ZBPYR. **(A)** Positive ion mode. **(B)** Negative ion mode.

The UHPLC-ESI-Q-TOF-MS total ion current chromatogram and results of ZBPYR are shown in Figure 1 and Supplementary Tables 1, 2. One hundred twenty-three compounds in the positive ion mode and 120 compounds in the negative ion mode were detected, and 75 compounds were found in both the positive ion and negative ion mode.

### ZBPYR Improved Glucose Metabolism Disorders in ZDF Rats

**FIGURE 2:**
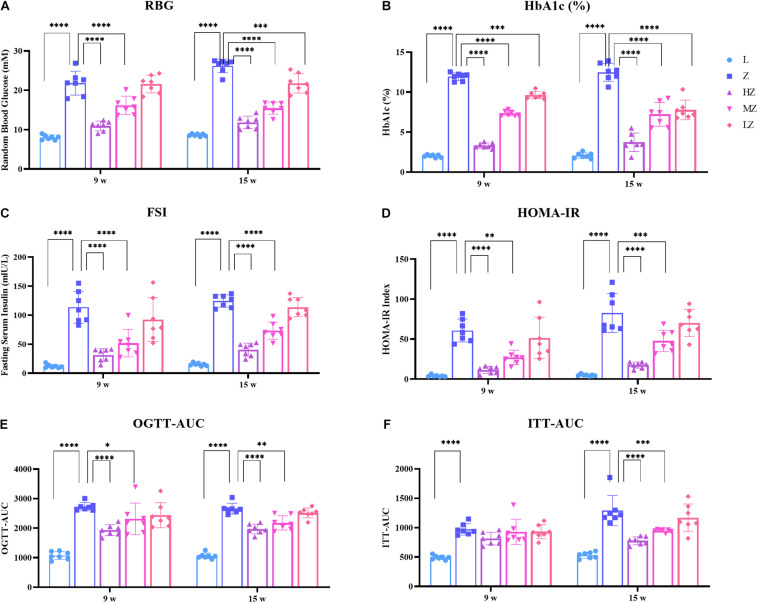
Effect of ZBPYR on the phenotype of glucose metabolism in rats. **(A)** Random blood glucose (RBG). **(B)** Glycosylated hemoglobin (HbA1c%). **(C)** Fasting serum insulin (FSI). **(D)** Homeostasis model of assessment for insulin resistance (HOMA-IR) index. **(E)** Area under the curve of the oral glucose tolerance test (OGTT-AUC). **(F)** Area under the curve of the insulin secretion test (ITT-AUC). Data are shown as mean ± SD. *n* = 7 per group. *p < 0.05, **p < 0.01, ***p < 0.001, ****p < 0.0001.

After 4 and 10 weeks of the experiment, ZDF rats gradually developed symptoms of glucose metabolism disorders. Specifically, ZDF rats showed increased levels of RBG, HbAc1%, FSI, and HOMA-IR, compared with the control group, respectively (*p* < 0.0001). High- and medium-dose ZBPYR significantly reduced the level of each index in a dose-dependent manner (*p* < 0.0001, *p* < 0.01, *p* < 0.001) (Figures 2A–D). Low-dose ZBPYR also reduced the level of HbAc1% in ZDF rats after 4 and 10 weeks of the experiment (*p* < 0.001, *p* < 0.0001) (Figure 2B). OGTT and ITT showed that ZDF groups had symptoms of impaired glucose tolerance and decreased insulin sensitivity after 4 and 10 weeks of the experiment (*p* < 0.0001) (Figures 2E,F). Both high- and medium-dose ZBPYR improved the condition of impaired glucose function (*p* < 0.0001, *p* < 0.05, *p* < 0.01) (Figure 2E), whereas the improvement in insulin intolerance only occurred after 10 weeks of the experiment (*p* < 0.0001, *p* < 0.001) (Figure 2F). The blood glucose levels of OGTT and ITT at each time point are shown in Supplementary Figures 1A,B. In addition, the BW and AC of rats in each group increased (Supplementary Figures 2A,B). After 4 weeks of the experiment, the BW and AC of the 9w-Z group were significantly higher than those of the 9w-L group (*p* < 0.0001) (Supplementary Figures 2A,B). In the ZBPYR high-dose treatment group, a decrease in BW and AC was observed (*p* < 0.05, *p* < 0.0001) (Supplementary Figures 2A,B). After 10 weeks of the experiment, high- and medium-dose ZBPYR reduced BW and AC in a dose-dependent manner (*p* < 0.0001, *p* < 0.001) (Supplementary Figures 2A,B). This indicated that ZBPYR ameliorated the disorder of glucose metabolism in ZDF rats.

### ZBPYR Improved Cognitive Dysfunction in ZDF Rats

**FIGURE 3:**
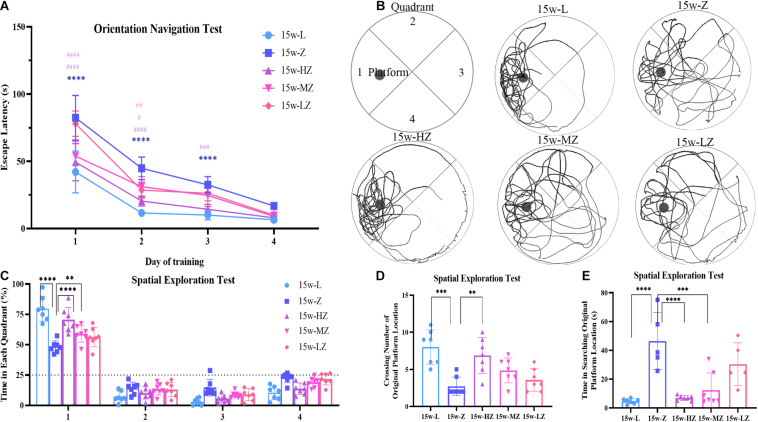
Morris water maze test was used to evaluate the effect of ZBPYR on learning and memory performance of rats. **(A)** The escape latency during a 4-day training course in the orientation navigation test. **(B–E)** Representative swimming path **(B)**, time spent in each quadrant (%) **(C)**, crossing number of the original platform location **(D)**, and time in searching original platform location **(E)** in the spatial exploration test. The small circle in Figure 2B represents the original platform location, but the escape platform was removed in the spatial exploration test. Data are shown as mean ± SD. *n* = 7 per group. ****p < 0.0001, compared with 15w-L group; ^#^*p* < 0.05, ^##^*p* < 0.01, ^###^*p* < 0.001, ^####^*p* < 0.0001, compared with 15w-Z group in Figure 2A. **p < 0.01, ***p < 0.001, ****p < 0.0001 in Figures 2C–E.

The MWM test was used to evaluate the learning and memory abilities of ZDF rats. In the orientation navigation test, with increased training times, the escape latency of rats gradually shortened (Figure 3A). From day 1 to day 3, the time to find the platform in the 15w-Z group was significantly longer than that in the 15w-L group (*p* < 0.0001), whereas the escape latency for the 15w-HZ group was significantly shorter than that of the 15w-Z group (*p* < 0.0001, *p* < 0.001) (Figure 3A). The escape latency of the 15w-MZ group and the 15w-LZ group was only different from the 15w-Z group from day 1 to day 2 (*p* < 0.0001, *p* < 0.05) (Figure 3A). After 4 days of training, rats have learned the location of the escape platform. In the spatial exploration test, rats in the 15w-Z group showed a marked decrease of time in the target quadrant (*p* < 0.0001) (Figure 3C) and crossing number of the original platform (*p* < 0.001) (Figure 3D) and an increase in searching time for the original platform (*p* < 0.0001) (Figure 3E), compared with the 15w-L group. After treatment with ZBPYR, ZDF rats showed a significant improvement in cognition function, especially in the high-dose treatment group (*p* < 0.0001, *p* < 0.01) (Figures 3C–E). The representative path of the rat during the exploration is shown in Figure 3B. During the orientation navigation test and spatial exploration test, there was no significant difference in the swimming speed of the rats (Supplementary Figures 3A,B), excluding the influence of physical ability and perceptual ability on spatial memory. As expected, ZBPYR significantly improved the cognitive function of ZDF rats in a dose-dependent manner.

Based on the above pharmacodynamic data, high-dose ZBPYR (HZ) had the best efficacy among all treatments according to various indexes. Thus, we next conducted comprehensive microbiomics and metabolomics analyses of samples from the L group (control group), Z group (model group), and HZ group (high-dose ZBPYR group).

### ZBPYR Modulated the Overall Composition of Gut Microbiota and Key Bacterial Species in ZDF Rats

**FIGURE 4:**
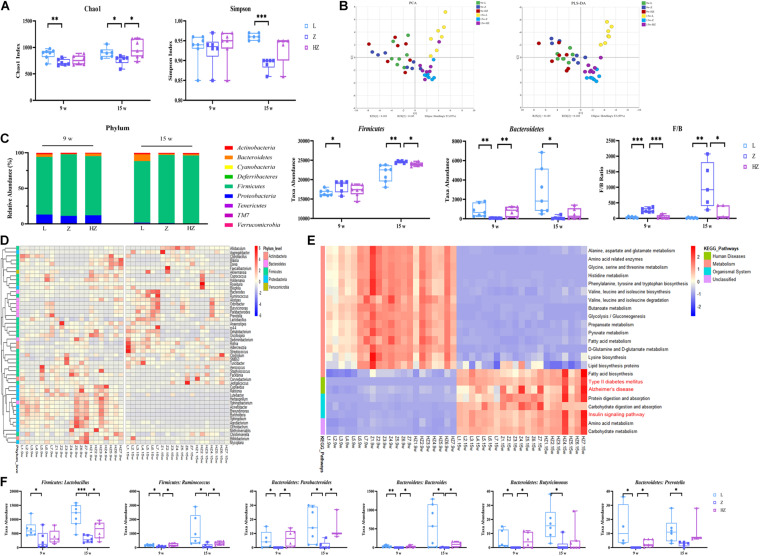
Effect of ZBPYR treatment on the gut microbiota of rats. **(A)** Chao 1 index and Simpson index. **(B)** Microbiota community analysis based on PCA and PLS-DA score plots. **(C)** Bacterial taxonomic composition profiling of gut microbiota at the phylum level. **(D)** Hierarchical clustering heatmap of the top 50 differentiated taxa at the genus level. **(E)** KEGG metabolic pathway prediction by PICRUSt analysis. **(F)** Abundance of the key differentiated taxa in different groups. *n* = 7 per group. *p < 0.05, **p < 0.01, ***p < 0.001. PCA, principal component analysis; PLS-DA, partial least squares discriminant analysis.

16S rRNA sequencing analysis was performed to explore DACD-associated differences in the bacterial community of the intestinal contents and to investigate the effects of ZBPYR on the microbiota community. The Chao 1 and Simpson index of the gut microbiota indicated no significant difference in α diversity among groups after 4 weeks of experiments (Figure 4A), whereas relatively few overall bacterial species were seen in the model groups, compared to the control and ZBPYR treatment groups after 10 weeks of experiments (*p* < 0.05, *p* < 0.001) (Figure 4A), indicating that the intervention of ZBPYR changes the abundance of bacterial communities. In the unweighted UniFrac PCA (*R*^2^*X* = 0.459, *Q*^2^ = 0.165) and PLS-DA (*R*^2^*X* = 0.463, *Q*^2^ = 0.382) score plot, these two groups were clearly separated into different clusters at 4 and at 10 weeks (Figure 4B). At the same time, the microbiota community structure of model group remarkably diverged from that of the control and ZBPYR treatment groups after 10 weeks of experiments, indicating that the bacterial community structure of ZDF rats gradually changed after ZBPYR treatment (Figure 4B). Phylum-level analysis revealed a marked temporal shift in the taxonomic distribution of the gut microbiota from T2DM to DACD, as evident from the dynamically altered abundance of the two dominant phyla *Firmicutes* and *Bacteroidetes* (Figure 4C). The 15w-Z group had a significantly higher abundance of *Firmicutes* and a significantly lower abundance of *Bacteroidetes*, compared with the 15w-L group (Figure 4C). The ratio of *Firmicutes* and *Bacteroidetes* populations is critical for the stability of gut microbiota, and the reduction of the ratio by ZBPYR may be the main reason for the gut microbiota changes (Figure 4C). The abundance of *Proteobacteria* in 15-week-old rats decreased after 10 weeks of experiments (Figure 4C). Heatmap shows the top 50 differentiated taxa with the highest genus level. The results demonstrated a distinct clustering of the composition of microbiota community composition for the model and control groups, and the ZBPYR treatment group was closer to the control group (Figure 4D). In addition, OPLS-DA analysis showed clear separation between the groups (Supplementary Figure 4). Genus-level profiling demonstrated that six species of bacteria had undergone dynamic changes during the development of DACD from T2DM. Among them, two genera (*Lactobacillus* and *Ruminococcus*) belong to *Firmicutes*, and four species (*Parabacteroides*, *Bacteroides*, *Butyricimonas*, and *Prevotella*) belong to *Bacteroidetes* (*p* < 0.05, *p* < 0.001, *p* < 0.01) (Figure 4F). Treatment with ZBPYR notably reversed the microbial dysbiosis in ZDF rats to control levels (Figure 4F). Of note, 23 altered functional characteristic pathways of KEGG were predicted from the 16S rRNA gene profile through PICRUSt analysis, namely, 10 amino acid metabolism pathways, 3 lipid metabolism pathways, 5 carbohydrate metabolism pathways, 2 human disease metabolic pathways, and 3 biological system pathways (Figure 4E). Hence, these findings indicated time-dependent dynamic alterations in the gut microbiota as DACD developed from T2DM, and the derivatives of *Firmicutes* and *Bacteroidetes* were the key distinguishing alterations for group separation. The change in the ZBPYR-regulated gut microbiota structure may be involved in the improvement in cognitive function.

### ZBPYR Affected Insulin Resistance and Amyloid Plaque Burden in the Brain of ZDF Rats

**FIGURE 5:**
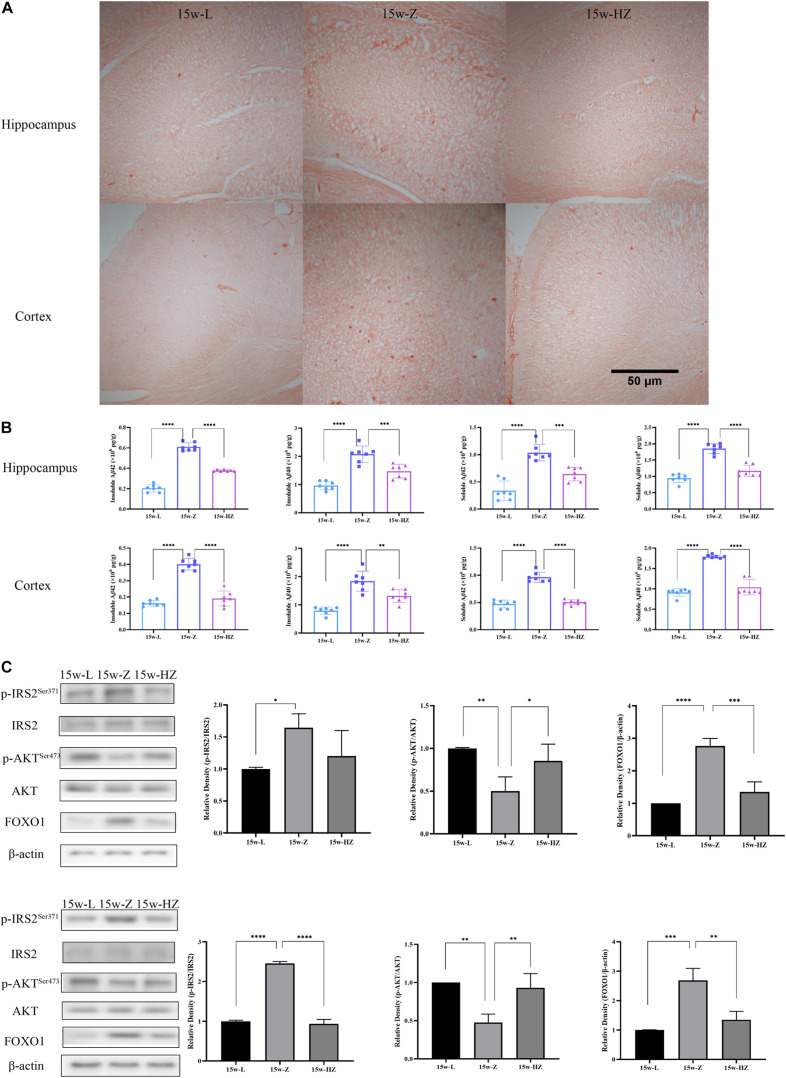
Effect of ZBPYR on Aβ deposition and insulin signaling pathway protein expression in the brain. **(A)** Congo red staining (40×). **(B)** Aβ content in hippocampus and cortex. **(C)** Protein expression of insulin signaling pathway in hippocampus and cortex. Data are shown as mean ± SD. *n* = 7 per group. *p < 0.05, **p < 0.01, ***p < 0.001, ****p < 0.0001.

Previous studies have shown that the hippocampus and cortex perform important functions for learning and memory formation. PICRUSt analysis showed that the Alzheimer’s disease (AD) and insulin signaling pathway were mainly enriched in 15-week-old ZDF rats. Therefore, we performed Congo red staining on hippocampus and cortex regions to detect compact amyloid plaques. The number of brick-red patches in the hippocampus and cortex of the 15w-Z group increased (Figure 5A), compared with the 15w-L group. The number of plaques in the 15w-HZ group was significantly lower than that in the 15w-Z group (Figure 5A), indicating that ZBPYR treatment could lead to a reduction in amyloid plaque burden. Aβ is the main component of amyloid plaques (Meng et al., 2020). Similarly, Aβ levels in the hippocampus and cortex of the 15w-Z group were significantly increased (*p* < 0.0001) (Figure 5B) compared with the 15w-L group. The level of Aβ in the 15w-HZ group was significantly lower than that in the 15w-Z group (*p* < 0.0001, *p* < 0.001, *p* < 0.01) (Figure 5B). These results suggested that the amyloid plaque burden in the brain of ZDF rats may be altered following treatment with ZBPYR. In addition, the expression of p-IRS2 and FOXO1 increased (*p* < 0.05, *p* < 0.0001, *p* < 0.001), and the expression of p-AKT decreased (*p* < 0.01) in the 15w-Z group, compared with the 15w-L group, confirming that insulin resistance appeared in the brain of 15-week-old ZDF rats (Figure 5C). ZBPYR treatment reversed the expression of these proteins (*p* < 0.05, *p* < 0.001, *p* < 0.0001) (Figure 5C), indicating that ZBPYR may improve brain insulin resistance in ZDF rats.

### ZBPYR Dynamically Restored the Global Metabolic Structure Abnormalities and Metabolic Alterations in ZDF Rats

**FIGURE 6:**
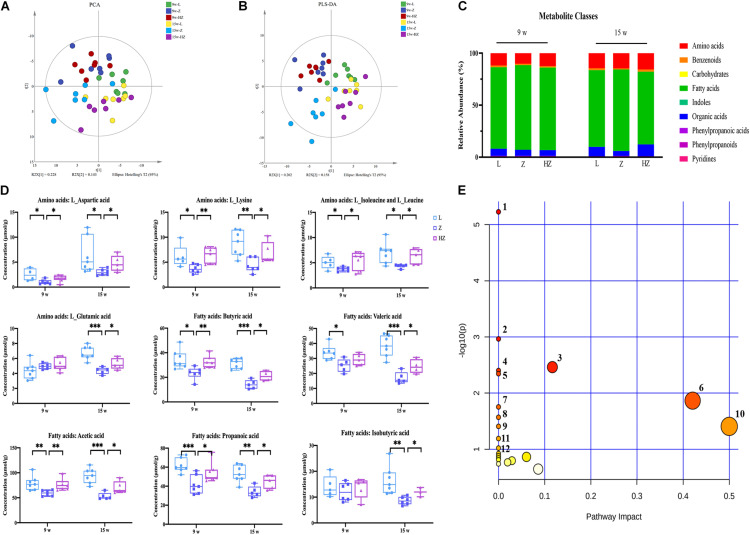
Effect of ZBPYR on the composition of metabolites in the intestinal contents of rats. **(A)** Global metabolite analysis based on PCA score plots. **(B)** Global metabolite analysis based on PLS-DA score plots. **(C)** Composition of metabolite classes analysis. **(D)** Differential metabolites in intestine. **(E)** Diagram of the metabolic enrichment pathways. *n* = 7 per group. *p < 0.05, **p < 0.01, ***p < 0.001. PCA, principal component analysis. PLS-DA, partial least squares discriminant analysis.

In order to obtain comprehensive metabolic profiles of the biological samples, targeted metabolomics profiling analysis was conducted using UPLC-MS/MS approaches, because it is more suitable for providing metabonomics-related information related to gut (Figures 6A,B) microbiota. A total of 103 metabolites (including amino acids, benzenoids, carbohydrates, fatty acids, indoles, organic acids, phenylpropanoic acids, phenylpropanoids, and pyridines) were ultimately identified and quantified (Figure 6C). Amino acids, fatty acids, and organic acids were the predominant types of annotated metabolites (Figure 6C). The PCA (*R*^2^*X* = 0.689, *Q*^2^ = 0.334) and PLS-DA (*R*^2^*X* = 0.516, *Q*^2^ = 0.420) score plot showed an aggregation tendency corresponding to 4 and 10 weeks of experiments, respectively (Figures 6A,B). A clear separation trend was seen between the control group and the model group after 10 weeks of experiments, indicating that the metabolic profiles of the intestinal contents from ZDF rats significantly varied from those of LZ rats (Figures 6A,B). Impressively, after 10 weeks of experiments, the intestinal content samples of ZBPYR treatment group showed a time-dependent dynamic trajectory that deviated from the model group (Figures 6A–C). Collectively, ZBPYR treatment significantly reversed the intestinal content metabolic profiles in ZDF rats over time.

Orthogonal partial least squares discriminant analysis was used to maximize category differentiation and identify potential biomarkers between groups. Metabolic profiles between the groups were completely separated, which reflected the different potential biomarkers that could distinguish them in OPLS-DA score plots (Supplementary Figure 5). VIP > 1 and *p* < 0.05 were used to identify differential metabolites in intestinal content samples. Ultimately, the levels of nine metabolites were found to be dynamically changed during the development of DACD from T2DM, namely, four types of amino acids (L-aspartic acid, L-lysine, L-isoleucine and L-leucine, and L-glutamic acid) and five types of SCFAs (butyric acid, valeric acid, acetic acid, propionic acid, and isobutyric acid) (*p* < 0.05, *p* < 0.01, *p* < 0.001) (Figure 6D). ZBPYR treatment improved the concentrations of amino acids and SCFAs in a time-dependent manner in ZDF rats (Figure 6D).

**FIGURE 7:**
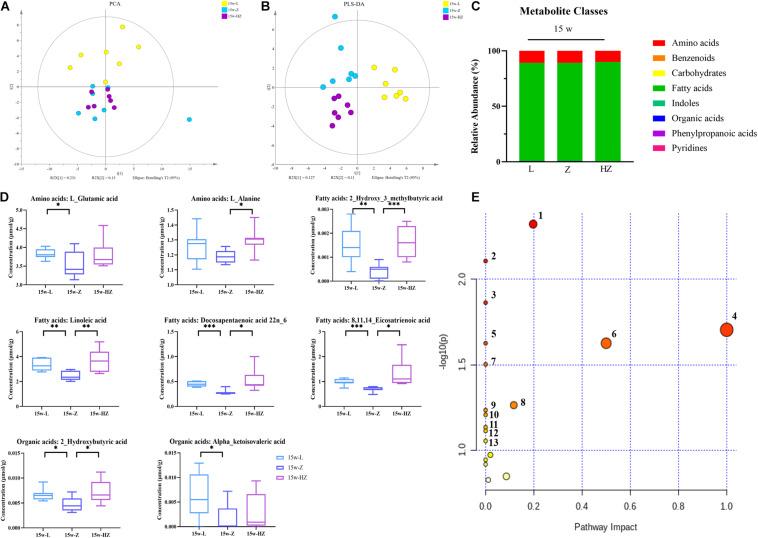
Effect of ZBPYR on the composition of metabolites in the hippocampus of rats. **(A)** Global metabolite analysis based on PCA score plots. **(B)** Global metabolite analysis based on PLS-DA score plots. **(C)** Composition of metabolite classes analysis. **(D)** Differential metabolites in hippocampus. **(E)** Diagram of metabolic enrichment pathways. *n* = 7 per group. *p < 0.05, **p < 0.01, ***p < 0.001. PCA, principal component analysis; PLS-DA, partial least squares discriminant analysis.

In addition, the metabolomic changes in the intestine may affect the metabolic structure of the brain *via* the microbiota–gut–brain axis, and brain metabolites contribute to the pathogenesis of DACD. Thus, in order to determine which metabolites might be mediators of the memory response, we also determined the levels of metabolites in the hippocampus of 15-week-old rats. We focused on the hippocampus, as it is an important area for acquiring and forming new memories (Reaven et al., 1990). Fewer metabolite differences were found between species in intestinal contents and hippocampus, with the exception of lower hippocampus levels of organic acids (Figure 7C). ZBPYR treatment changed the brain metabolite structure of ZDF rats (Figures 7A,B and Supplementary Figure 6). In the hippocampus, eight metabolites were significantly lower in the 15w-Z group, namely, two types of amino acids (L-glutamic acid and L-alanine), four types of fatty acids [2-hydroxy-3-methylbutyric acid, linoleic acid, docosapentaenoic acid (22n-6), and 8,11,14-eicosatrienoic acid], and two types of organic acids (2-hydroxybutyric acid and alpha-ketoisovaleric acid), compared with the 15w-L group (*p* < 0.05, *p* < 0.01, *p* < 0.001) (Figure 7D). ZBPYR treatment significantly reversed all these differential metabolites (Figure 7D), indicating that evaluating the potential application of these differential metabolites in the diagnosis of DACD has good diagnostic accuracy. The potential differential metabolites and their detailed information are listed in Supplementary Tables 3, 4.

All potential differential metabolites were imported into the web-based MetaboAnalyst 4.0 system for pathway analysis. Of note, the potential biomarkers of intestinal contents mainly involved 12 affected metabolic pathways (*p* < 0.1) (Figure 6E and Table 2), and the potential biomarkers of hippocampus mainly involved 13 metabolic pathways (*p* < 0.1) (Figure 7E and Table 3). The common enrichment pathways of intestinal contents and hippocampus metabolites were four amino acid metabolism pathways and one carbohydrate metabolism pathway. Three amino acid metabolism pathways (alanine, aspartic acid, and glutamate metabolism; valine, leucine, and isoleucine biosynthesis; and D-glutamine and D-glutamate acid metabolism) and one carbohydrate metabolism pathway (butyrate metabolism) were identified, consistent with the predicted results of PICRUSt, which verifies the reliability of the results.

**TABLE 2:** Metabolite pathway changes in intestine.

No.	Pathway	Total	Hits	Raw *p*	Log(*p*)	Holm *p*	FDR	Impact
1	Aminoacyl-tRNA biosynthesis	48	5	0.00	5.23	0.00	0.00	0.00
2	Valine, leucine, and isoleucine biosynthesis	8	2	0.00	2.96	0.09	0.05	0.00
3	Arginine biosynthesis	14	2	0.00	2.46	0.28	0.08	0.12
4	Butanoate metabolism	15	2	0.00	2.40	0.32	0.08	0.00
5	Histidine metabolism	16	2	0.00	2.35	0.36	0.08	0.00
6	Alanine, aspartate, and glutamate metabolism	28	2	0.01	1.87	1.00	0.19	0.42
7	Glyoxylate and dicarboxylate metabolism	32	2	0.02	1.75	1.00	0.21	0.00
8	Valine, leucine, and isoleucine degradation	40	2	0.03	1.57	1.00	0.28	0.00
9	Nitrogen metabolism	6	1	0.04	1.41	1.00	0.33	0.00
10	D-Glutamine and D-glutamate metabolism	6	1	0.04	1.41	1.00	0.33	0.50
11	Biotin metabolism	10	1	0.06	1.19	1.00	0.49	0.00
12	Nicotinate and nicotinamide metabolism	15	1	0.10	1.02	1.00	0.67	0.00

*Hits represent the matched number of metabolites in the pathway. Raw p represents the original p-value calculated from the enrichment analysis. Holm p represents the p-value further adjusted using the Holm–Bonferroni method. FDR (false discovery rate) represents the p-value adjusted using false discovery rate.*

**TABLE 3:** Metabolite pathway changes in hippocampus.

No.	Pathway	Total	Hits	Raw *p*	Log(*p*)	Holm adjust	FDR	Impact
1	Aminoacyl-tRNA biosynthesis	48	2	0.01	1.86	1.00	0.33	0.00
2	Alanine, aspartate, and glutamate metabolism	28	2	0.00	2.32	0.40	0.33	0.20
3	Histidine metabolism	16	1	0.06	1.21	1.00	0.52	0.00
4	Valine, leucine, and isoleucine biosynthesis	8	1	0.03	1.50	1.00	0.38	0.00
5	Butanoate metabolism	15	1	0.06	1.23	1.00	0.52	0.00
6	Propanoate metabolism	23	1	0.09	1.05	1.00	0.57	0.00
7	Nitrogen metabolism	6	1	0.02	1.63	1.00	0.33	0.00
8	Biosynthesis of unsaturated fatty acids	36	2	0.01	2.11	0.65	0.33	0.00
9	Linoleic acid metabolism	5	1	0.02	1.70	1.00	0.33	1.00
10	Pantothenate and CoA biosynthesis	19	1	0.07	1.13	1.00	0.54	0.00
11	D-Glutamine and D-glutamate metabolism	6	1	0.02	1.63	1.00	0.33	0.50
12	Selenocompound metabolism	20	1	0.08	1.11	1.00	0.54	0.00
13	Arginine biosynthesis	14	1	0.05	1.26	1.00	0.52	0.12

*Hits represent the matched number of metabolites in the pathway. Raw p represents the original p-value calculated from the enrichment analysis. Holm p represents the p-value further adjusted using the Holm–Bonferroni method. FDR (false discovery rate) represents the p-value adjusted using false discovery rate.*

### Bioinformatics Analysis and Correlation Network Construction of the Microbiota–Gut–Brain Axis-Based Mechanism of ZBPYR Against DACD

**FIGURE 8:**
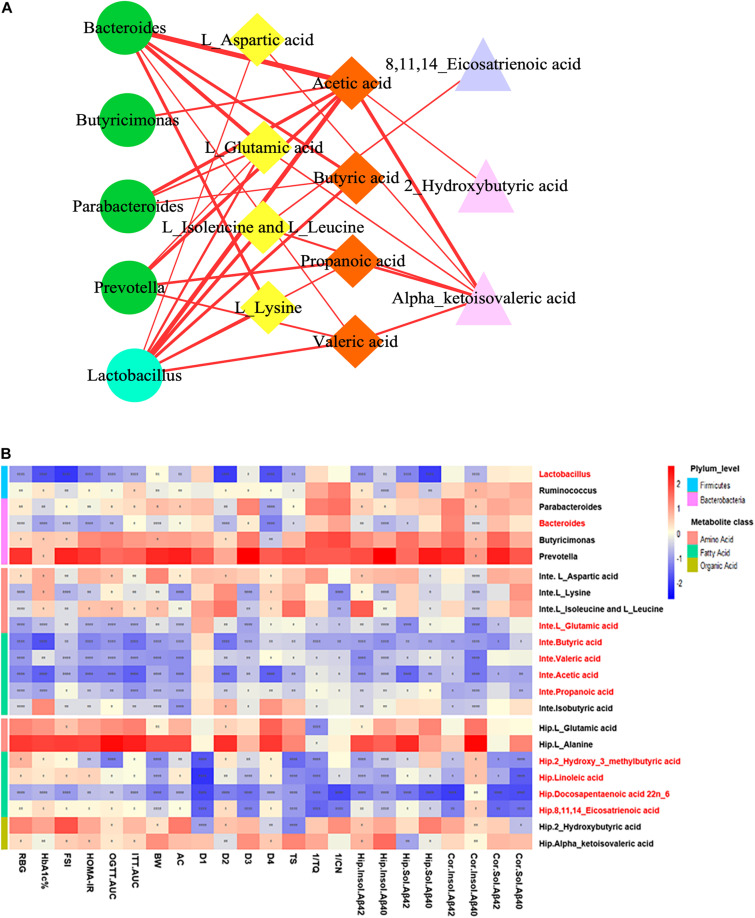
Correlation analysis integrating bacterial genera, intestinal metabolites, hippocampus metabolites, and DACD phenotypes. **(A)** Network diagram showing the correlation among bacterial genera (green circle denotes *Bacteroidetes*; cyan circle indicates *Firmicutes*), intestinal metabolites (yellow rhombus denotes aliphatic amino acids; orange rhombus indicates short chain fatty acids), and hippocampus metabolites (purple triangle denotes unsaturated fatty acids; pink triangle indicates aliphatic organic acids). **(B)** Heatmap showing the correlation between potential biomarkers and DACD phenotype. The degree of correlation is shown by the gradient change of red (positive correlation) and blue (negative correlation). *p < 0.05, **p < 0.01, ***p < 0.001, ****p < 0.0001. RBG, random blood glucose; HbA1c%, glycosylated hemoglobin; FSI, fasting serum insulin; HOMA-IR, homeostasis model of assessment for insulin resistance; OGTT-AUC, area under the curve of the oral glucose tolerance test; ITT-AUC, area under the curve of the insulin secretion test; BW, body weight; AC, abdominal circumference; D1–D4, escape latency during a 4-day training course in the orientation navigation test of Morris water maze test; TS, time in searching original platform location in the spatial exploration test of Morris water maze test; 1/TQ, the reciprocal of time spent in each quadrant (%) in the spatial exploration test of Morris water maze test; 1/CN, the reciprocal of crossing number of the original platform location in the spatial exploration test of Morris water maze test; Inte, intestine; Hip, hippocampus; Cor, cortex.

**FIGURE 9:**
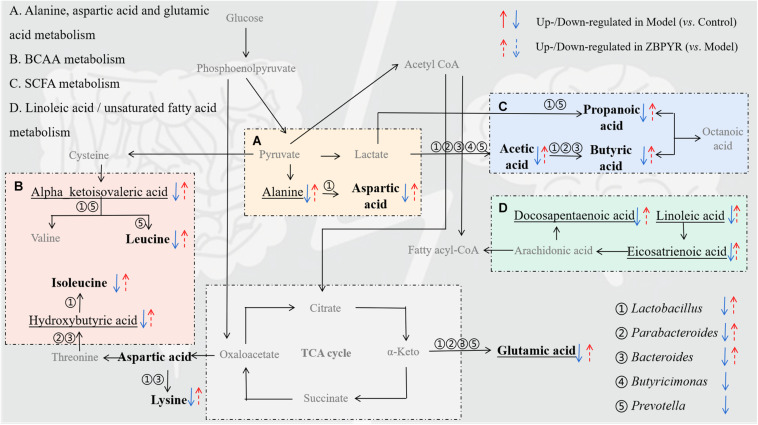
Schematic diagram showing the regulation of ZBPYR treatment on the network of differentiated bacterial and metabolite structural components. **(A–D)**, enriched metabolic pathways; ①–⑤, differential bacterial genera; bold, differential intestinal metabolites; underline, differential hippocampus metabolites. BCAA, branched chain amino acid. SCFA, short-chain fatty acid.

To explore the functional relationship of the altered intestinal bacterial genera and the disrupted metabolites in DACD, Spearman correlation analysis was performed. Network diagram shows the relationship among five different bacterial genera (*Ruminococcus* is not shown, *p* > 0.05) and 17 metabolites (nine intestinal metabolites and eight hippocampus metabolites) (Figure 8A). In the correlation between intestinal bacterial genera and intestinal metabolites, *Lactobacillus* was positively correlated with almost all intestinal metabolites, and almost all bacterial genera were positively correlated with L-glutamic acid and acetic acid; *Bacteroides* was positively correlated with L-lysine, butyric acid, and valeric acid; *Parabacteroides* was positively correlated with butyric acid; *Prevotella* was positively correlated with propionic acid and valeric acid. In the correlation between intestinal metabolites and hippocampus metabolites, alpha-ketoisovaleric acid was positively correlated with almost all intestinal metabolites; 2-hydroxybutyric acid was positively correlated with acetic acid; 8,11,14-eicosatrienoic acid was positively correlated with L-leucine and L-isoleucine. Moreover, a heatmap shows the correlation between potential biomarkers and DACD phenotype (Figure 8B). In all correlation analyses, *Lactobacillus* and *Bacteroides* among bacterial genera, acetic acid, propionic acid, butyric acid, valeric acid, and L-glutamic acid in intestinal metabolites; and 2-hydroxy-3-methylbutyric acid, 8,11,14-eicosatrienoic acid, docosapentaenoic acid 22n-6, and linoleic acid in hippocampus metabolites were significantly negatively correlated with DACD-related phenotypes. Following the integration of bioinformatics search and correlation analysis, the potential network of key DACD-related microbiota species and differentiated metabolites was depicted (Figure 9). The related metabolite biomarkers of DACD disrupted by ZBPYR were mainly involved in the following pathways: alanine, aspartic acid, and glutamic acid metabolism; branched chain amino acid (BCAA) metabolism; SCFA metabolism; and linoleic acid/unsaturated fatty acid metabolism. As the critical axis elements in the “microbiota–gut–brain axis,” metabolites can enter the circulation and affect the communication between the intestine and the brain. Therefore, in our present study, these biomarkers were predicted as a potential “microbiota–gut–brain axis,” which may contribute to the development of DACD and the therapeutic targets of ZBPYR.

## Discussion

It is speculated that the gut microbiota plays an important role in regulating the metabolic pathways of the host and plays an important role in a variety of disease phenotypes (He W. et al., 2020; Leclercq et al., 2020; Ling et al., 2020; Shi et al., 2020b; van Soest et al., 2020). In recent years, people have realized that the microbiota–gut–brain axis is a novel concept in the field of neurological disease research (Li et al., 2020; Mao et al., 2020; Shi et al., 2020a). Strong lines of evidence have suggested that TCM plays an important role in regulating the gut microbiota in various complex diseases (Du et al., 2020). Our previous studies have indicated that ZBPYR may ameliorate the metabolic phenotype of T2DM and DACD, and exhibit a potential effect on regulating the disturbance of probiotics and pathogenic bacteria in gut microbiota (Gu et al., 2017). However, it still remains unclear that whether microbiota-gut-brain axis which mediated by ZBPYR have a potential role in the development of DACD. It is a challenge to clarify the mechanism of action of TCM, which is the result of the unknown synergy of its complex components. In addition, a single microbiomics or metabolomics strategy cannot fully illustrate the pathophysiological process of DACD (Bo et al., 2020). Both TCM and omics have common views of integrity, individuality, and dynamics. Thus, we sought to determine the relationship between the efficacy of ZBPYR in preventing and treating DACD and the key microbiome and metabolome regulation involved in DACD progression over time.

In recent years, the CNS complications of T2DM have gained considerable attention, and the molecular mechanism of related learning and memory has made great progress (Zheng et al., 2017; Ma et al., 2020). The present results indicated that ZDF rats developed signs of T2DM at 9 weeks old, and prominent DACD-related cognitive decline at 15 weeks old, as expected. T2DM symptoms and cognitive behavior of ZBPYR-treated ZDF rats tended to be normal, and Aβ deposition and insulin resistance in the brain were also reversed, indicating that ZBPYR had a certain preventive and therapeutic effect on DACD. Interestingly, ZBPYR could remarkably restore the DACD-altered microbiota compositions in a time-dependent manner in ZDF rats, produced lasting changes in gut microbiota community and metabolites, and remotely affected hippocampus metabolic changes, thereby affecting cognitive behavior. Among them, anshinone and danshensu in Dan-Shen, paeoniflorin in Bai-Shao, and the volatile oil components such as β-asarone in Shi-Chang-Pu can directly cross the blood–brain barrier (BBB) to improve cognitive impairment (Sun et al., 2020). In addition, the main active ingredients in ZBPYR, such as ginsenosides in Hong-Shen (Sun et al., 2020), yam polysaccharides in Shan-Yao (Pang et al., 2020), and saponins in Yuan-Zhi (Zeng et al., 2021), cannot directly cross the BBB. It is necessary to transform the metabolism of macromolecular substances into small molecular metabolites through the mediation of the gut microbiota and then cross the BBB to play a biological effect of improving cognitive function. We also provided metabolic evidence of links among the host, the gut microbiota, and memory responses. To our knowledge, this is the first evidence showing that cognitive functions of the CNS are significantly affected during the progression of DACD and that ZBPYR is related to a series of microbiota–gut–brain axis events in ZDF rats.

Healthy gut microbiota is very important for maintaining normal brain development and function (Tooley, 2020). More and more lines of evidence support the idea that the transfer of healthy microbiota improves learning and memory ability in different cognitive impairment models, providing a proof-of-concept illustration of the microbiota–gut–brain axis. In the present study, the distribution of the gut microbiota in 15-week-old ZDF rats was different from that in 9-week-old rats, indicating that the gut microbiota homeostasis of ZDF rats is imbalanced during the progression of DACD. Note that gut microbial dysbiosis is closely related to the reduction of phylogenetic abundance differences, especially in metabolic and neurological diseases (Liu et al., 2019). The underlying pathological basis of DACD microbiota appears to involve a shifting environment that is more conducive for the growth of detrimental bacteria and less conducive for the growth of beneficial bacteria. 16S rRNA sequencing results showed that the abundance of *Firmicutes* was significantly increased, and the abundance of *Bacteroidetes* was significantly decreased during the progression of DACD, which is consistent with changes in patients with T2DM and cognitive impairment. In addition, ZBPYR treatment affected the abundance of some key bacterial genera, such as *Lactobacillus*, *Ruminococcus*, *Parabacteroides*, *Bacteroides*, *Butyricimonas*, and *Prevotella*. We speculate that changes in these genera may be the main reason for the changes in cognitive function. It was reported that *Lactobacillus* improved cognitive ability in AD patients and AD rats induced by Aβ42 injection, which may be related to its ability to acidify the intestinal environment, produce essential amino acids, maintain barrier integrity, and reduce β-amyloid protein deposition in the brain (Mao et al., 2020). *Parabacteroides* is an anti-inflammatory bacterium that produces SCFAs. Current research has not yet combined its connection with human cognitive impairment. However, it has previously been demonstrated that transplanting gut microbiota of healthy rats into a T2DM model can increase the number of intestinal *Parabacteroides*. *Ruminococcus* is involved in certain gastrointestinal diseases and metabolic diseases in the intestine (Gomez-Arango et al., 2016; Jia et al., 2020), and recent studies have confirmed that it may participate in the process of cognitive dysfunction (Park et al., 2017). In addition, *Bacteroides*, *Butyricimonas*, and *Prevotella* have been confirmed to have reduced abundance in animal models or patients with cognitive impairment (Panee et al., 2018; Qian et al., 2018; Maldonado et al., 2019). These changes in the structure of gut microbiota indicate that ZBPYR treatment has an obvious effect on improving the pathological bacteria in ZDF rats during the pathogenesis of DACD.

Metabolomics information is the final result of biological functions and can directly indicate abnormal physiological conditions, because it is “downstream” of initial changes that occur at the genome, transcriptome, and proteome levels (Huang et al., 2016). The mammalian brain is characterized by high metabolic activity with accordance regulatory mechanisms to ensure the adequate supply of energy substrates in register with brain activity. We focused on understanding the metabolic mechanism behind the flow of metabolites from intestine to brain for memory improvement. Consistent with previous studies, the metabolic pathways enriched by DACD are primarily involved in amino acid metabolism and fatty acid metabolism (Zhang et al., 2018). Amino acid metabolism directly affects the activity of the nervous system (Arnoriaga-Rodriguez et al., 2020). For example, we observed an increase in L-glutamic acid and L-aspartic acid following ZBPYR treatment, which are neuron-specific markers for monitoring neuronal damage associated with CNS diseases, although they lack specificity for any particular disease (Paglia et al., 2016). Glutamate is an excitatory neurotransmitter, well known for its critical role in learning and memory, especially in the promotion of synaptic transmission (Chater and Goda, 2014) and induction and maintenance of long-term potentiation in hippocampus neurons (Miyamoto, 2006). Glutamate also contributes to inhibitory GABAergic regulation in the brain (Pereira et al., 2008). It has been confirmed that the abnormal GABA level in the brain of T2DM patients may be associated with the impairment of cognitive function (van Duinkerken et al., 2012). In this study, L-glutamic acid level was significantly downregulated in both intestine and hippocampus of ZDF rats, indicating the altered glutamate signaling (Divito and Underhill, 2014). Similar alterations in glutamate signaling have been reported in rodent models of AD. Glutamatergic dysfunction in the hippocampus and cortex has been identified as an important mediator of impaired cognition, which acts as an excitatory neurotransmitter. It plays a key role in learning and memory, especially in promoting synaptic transmission and maintaining the morphology and function of hippocampal neurons (Javitt, 2010; Nilsen et al., 2014). Aspartic acid, another excitatory neurotransmitter, is directly derived from transamination of the TCA cycle intermediate, oxaloacetate, and is closely related to cognitive decline. We found that the concentration of L-aspartic acid was decreased in ZDF rats, which is consistent with the decrease in TCA cycle activity observed in db/db mice with cognitive decline (Zheng et al., 2017). Alanine is not only a potential neurotransmitter at glycine receptors, but also a component of carnosine and an inhibitor of taurine transport. Evidence has shown that a disturbance of lactate-alanine shuttle in the hippocampus may be responsible for nitrogen exchange (Zwingmann et al., 2000; Schousboe et al., 2003) and related to the recovery of spatial memory (Sase et al., 2013; Han et al., 2017) in mammals. BCAAs are essential amino acids with branched aliphatic side chains and account for almost one-third of all amino acids in the human body (Wang et al., 2019). Three known proteinogenic BCAAs, valine, leucine, and isoleucine, cross the BBB and participate in the structure components and systemic function of brain tissue (Oldendorf, 1971). In addition, BCAAs are involved in neurotransmitter metabolism (glutamate metabolism); affect amyloid plaque load, neuron survival rate, and adult hippocampus neurogenesis; and significantly influence the entire function of the CNS (Fernstrom, 2005; Hawkins and Vina, 2016; Polis and Samson, 2020). In this study, we observed changes in the levels of leucine and isoleucine in the intestinal contents of ZDF rats, and changes in the levels of upstream metabolites hydroxybutyric acid and ketoisovaleric acid in the hippocampus. The improvement in cognitive dysfunction in ZDF rats may be a long-range effect of intestinal metabolites. Studies have confirmed that *Lactobacillus* can affect the level of amino acids by regulating the BCAA metabolic pathway (Wang et al., 2019). Lipids are important components of the brain and play a crucial role in cell signaling and various physiological processes (Saxena, 2009; Fraser et al., 2010; Zhang Q. et al. 2020). As a member of lipids family, SCFAs are associated with brain function and neurobiological effects in systemic circulation, which may mitigate Aβ deposition and insulin resistance in the brain (Sampson et al., 2016). In our study, ZBPYR treatment significantly increased SCFAs, including acetic acid, butyric acid, isobutyric acid, propanoic acid, and valeric acid, in the intestinal contents of ZDF rats, and the abundance of related bacterial genera that mainly produce SCFAs also increased, including *Parabacteroides*, *Prevotella*, and *Ruminococcus* (den Besten et al., 2013; La Reau and Suen, 2018). Among them, acetic acid (Shin et al., 2011) and butyric acid (Stilling et al., 2016) are known for their beneficial effects on neuronal health and cognitive function. In addition, studies have provided evidence linking DACD to disorders of linoleic acid metabolism (Beydoun et al., 2007; Chu et al., 2016). Linoleic acid is an important polyunsaturated fatty acid, and the anti-oxidative, anti-inflammatory, and anti-apoptotic effects at high concentrations in the brain may lead to neuron protection in nervous system diseases (Beydoun et al., 2007; Ramsden et al., 2012). Docosahexaenoic acid is an essential omega-3 long-chain polyunsaturated fatty acid (LCPUFA) that is important in the development of the nervous system. It has been shown to be involved in neurotransmitter conduction, signal transduction, and neurogenesis in the brains of T2DM patients, and has anti-inflammatory and other important functions (Echeverria et al., 2017). Collectively, the current lines of evidence support the hypothesis that the regulatory effects of gut metabolism-related amino acid metabolism and fatty acid metabolism in ZDF rats may be an important anti-DACD mechanism of ZBPYR. Although these differences cannot be explained at present, metabolomic analysis of the brain combined with metabolomic analysis of the gut microbiota and the circulatory interface between intestine and brain are likely to become a powerful tool to analyze microbiota–gut–brain axis communication in health and disease.

Gut microbiota may contribute to brain diseases; in particular, new connections between gut microbiota and cognitive functions have been reported in humans and animals. We found that ZDF rats show spatial learning and memory impairment, therefore enabling us to confirm that the cognitive deficits are the result of T2DM phenotype (such as chronic hyperglycemia and insufficient insulin secretion). In addition to its peripheral role in glucose utilization and insulin sensitivity, insulin is essential for multiple central processes, including information processing that is critical for cognition. In the process of memory encoding and retrieval, insulin receptor signaling can regulate the transmission of glutamatergic (enhanced through NMDA receptors) and GABAergic (recruited to the postsynaptic membrane through GABA receptors), thus regulating synaptic plasticity in the hippocampus and affecting cognitive ability (Soto et al., 2018). With differential species correlation analysis with the metabolic pathways, we observed that the underlying mechanism may be related to AD pathway and insulin signaling pathway. Conceivably, cognitive impairment due to persistent hyperglycemia in patients with T2DM may cause changes in bacterial metabolites, which stimulate insulin resistance, resulting in augmentation of Aβ pathology in the brain (Baluchnejadmojarad et al., 2017; Bi et al., 2019). Brain Aβ deposition is a hallmark of DACD and AD pathogenesis, as well as an important cause of cognitive decline (Sui et al., 2017). Recent studies have also confirmed the similarities between the changes seen in ZDF rats and brain samples from people with AD, namely, Aβ accumulation and insulin resistance in the brain causing neuronal injury and ultimately cognitive decline (Bi et al., 2020; Jash et al., 2020). The insulin-dependent IRS2-AKT signaling pathway is the main pathway of insulin resistance (Zhang L. et al. 2020). IRS2 and AKT are associated with neuronal growth and memory. Intraventricular injection of Aβ induces brain insulin resistance, as evidenced by hyperphosphorylation of IRS2 and AKT. Obvious changes in pathological lesions found in our current study were consistent with changes in learning and memory function, and ZBPYR reversed related protein expression. These findings were similar to previous findings showing that increased phosphorylation of IRS induced by DACD may decrease hippocampus and cortex insulin sensitivity. The brain with DACD including pathology of Aβ and associated insulin signaling pathway shows cross-talk with the intestine *via* the microbiota–gut–brain axis. Thus, ZBPYR may be a novel prevention and treatment strategy for regulating gut microbiota, ameliorating brain insulin resistance, and preventing early-stage pathologies associated with DACD.

## Conclusion

In summary, we characterized memory deficits during the pathogenesis of ZDF rats with T2DM that developed DACD and showed that these deficits were correlated with dysfunction in gut microbiota and altered microbiota metabolites in the hippocampus. Interestingly, we found that ZBPYR regulated DACD-related gut microbiota dysbiosis and reversed Aβ deposition and insulin resistance in the brain. These features may be related to a series of metabolic changes affected by gut microbiota, including alanine, aspartic acid, and glutamic acid metabolism; BCAA metabolism; SCFA metabolism; and linoleic acid/unsaturated fatty acids metabolism, although further investigation is needed. To our knowledge, this study is the first report to show that gut microbiota and its metabolic activity, as well as the efficacy of TCM, such as ZBPYR, may be related to the progression of DACD. There are good reasons to believe that the microbiota–gut–brain may provide a novel perspective to explore DACD-related pathogenic changes and facilitate a better understanding of TCM-based therapeutic avenues for preventing the development of DACD.

## Data Availability Statement

The datasets presented in this study can be found in online repositories. The names of the repository/repositories and accession number(s) can be found in the article/Supplementary Material.

## Ethics Statement

The animal experiment protocol was designed to minimize the pain or discomfort of animals and has been approved by the Animal Ethics Committee of Nanjing University of Chinese Medicine (permit number: 201901A009).

## Author Contributions

LZ and XL conceived the idea and designed the study. TB, RF, and WR performed the experiments, obtained the samples, and acquired the data. TB performed the microbiomics and metabolomics data analysis, conducted molecular biology experiments, and wrote the manuscript. LZ directed the project and was involved in modifying the manuscript. All authors had approved the final manuscript for submission.

## Conflict of Interest

The authors declare that the research was conducted in the absence of any commercial or financial relationships that could be construed as a potential conflict of interest.

## Publisher’s Note

All claims expressed in this article are solely those of the authors and do not necessarily represent those of their affiliated organizations, or those of the publisher, the editors and the reviewers. Any product that may be evaluated in this article, or claim that may be made by its manufacturer, is not guaranteed or endorsed by the publisher.

## Supplementary Material

Supplementary Figure 1 OGTT and ITT in rats. **(A)** OGTT measured in different groups. **(B)** ITT measured in different groups. Data are shown as mean ± SD. *n* = 7 per group. ^∗∗^*P* < 0.01, ^∗∗∗^*P* < 0.001, ^****^*P* < 0.0001, compared with the 15w-L group; ^#^*P* < 0.05, ^##^*P* < 0.01, ^###^*P* < 0.001, ^####^*P* < 0.0001, compared with the 15w-Z group. OGTT, oral glucose tolerance test; ITT, insulin secretion test.

Supplementary Figure 2 BW and AC in rats. **(A)** BW measured in different groups. **(B)** AC measured in different groups. Data are shown as mean ± SD. *n* = 7 per group. ^∗^*P* < 0.05, ^∗∗∗^*P* < 0.001, ^****^*P* < 0.0001. BW, body weight; AC, abdominal circumference.

Supplementary Figure 3 Swimming speed in Morris water maze test of the orientation navigation test and the spatial exploration test. Data are shown as mean ± SD. *n* = 7 per group. D, day.

Supplementary Figure 4 OPLS-DA score plots for the different groups in the intestinal contents microbiology of rats. OPLS-DA, orthogonal partial least squares discriminant analysis.

Supplementary Figure 5 OPLS-DA score plots for the different groups in the intestinal contents metabolomics of rats. OPLS-DA, orthogonal partial least squares discriminant analysis.

Supplementary Figure 6 OPLS-DA score plots for the different groups in the hippocampus metabolomics of rats. OPLS-DA, orthogonal partial least squares discriminant analysis.
