# Supplementary figures and images for: ZiBuPiYin Recipe Prevented and Treated Cognitive Decline in ZDF Rats With Diabetes-Associated Cognitive Decline via Microbiota–Gut–Brain Axis Dialogue
Source: Front Cell Dev Biol. 2021 Aug 18;9:651517. doi: 10.3389/fcell.2021.651517 (PMC8416319; doi:10.3389/fcell.2021.651517)

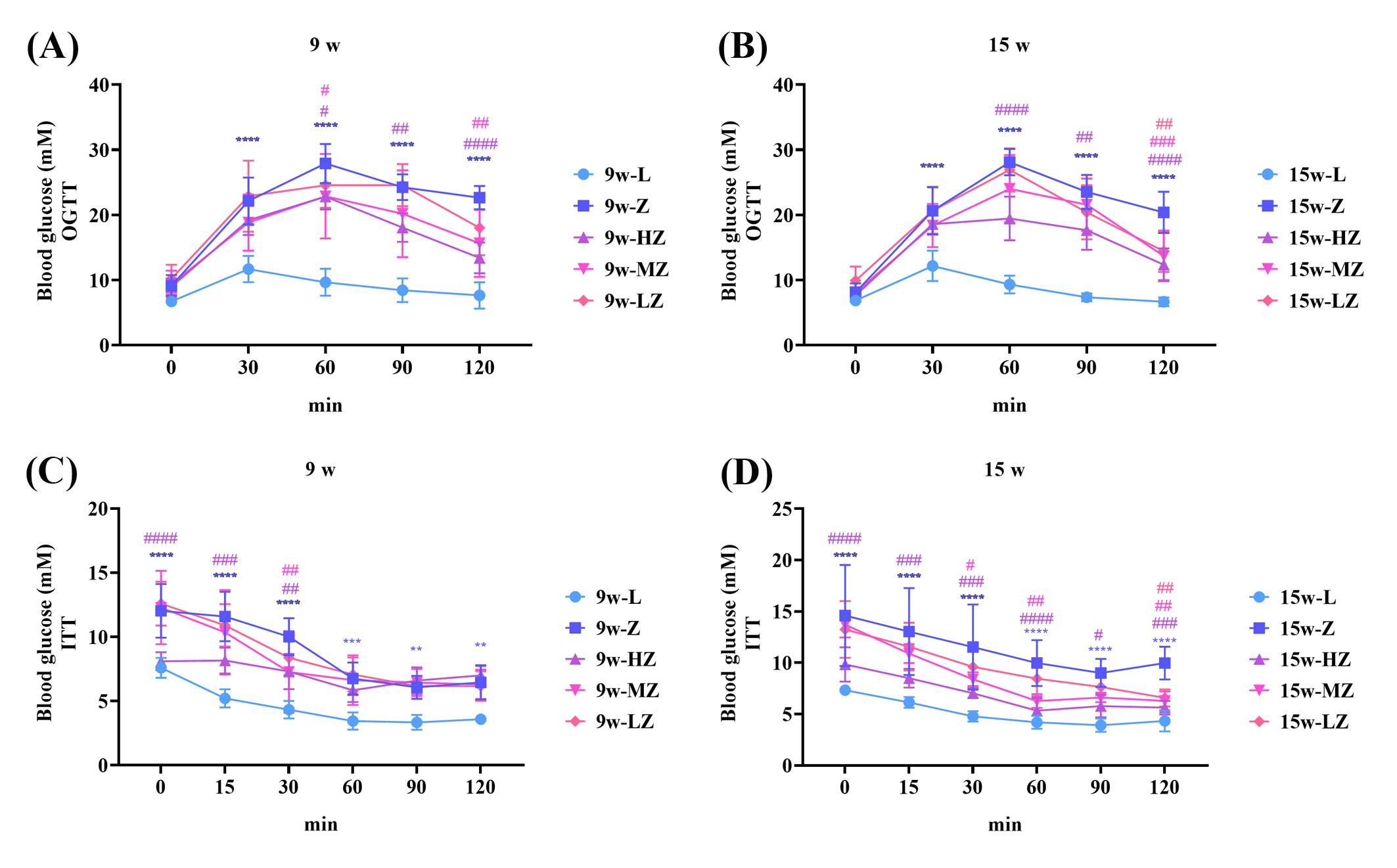

Supplement: Supplementary Figure 1 — OGTT and ITT in rats. (A) OGTT measured in different groups. (B) ITT measured in different groups. Data are shown as mean ± SD. n = 7 per group. ∗∗P < 0.01, ∗∗∗P < 0.001, ****P < 0.0001, compared with the 15w-L group; #P < 0.05, ##P < 0.01, ###P < 0.001, ####P < 0.0001, compared with the 15w-Z group. OGTT, oral glucose tolerance test; ITT, insulin secretion test. [file Image_1.TIF]

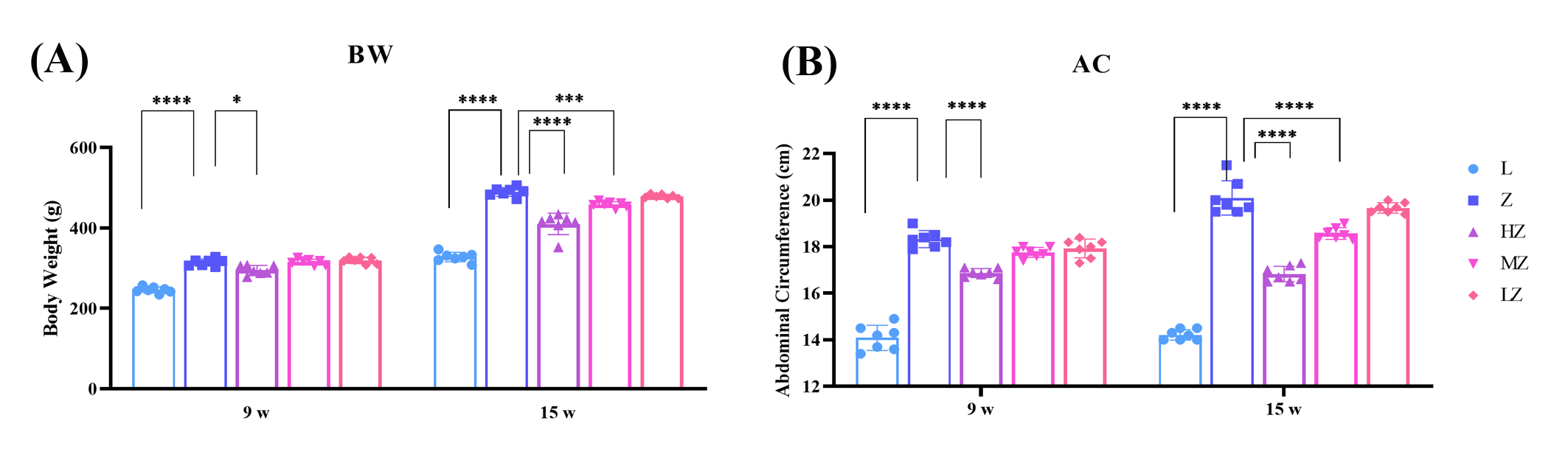

Supplement: Supplementary Figure 2 — BW and AC in rats. (A) BW measured in different groups. (B) AC measured in different groups. Data are shown as mean ± SD. n = 7 per group. ∗P < 0.05, ∗∗∗P < 0.001, ****P < 0.0001. BW, body weight; AC, abdominal circumference. [file Image_2.TIF]

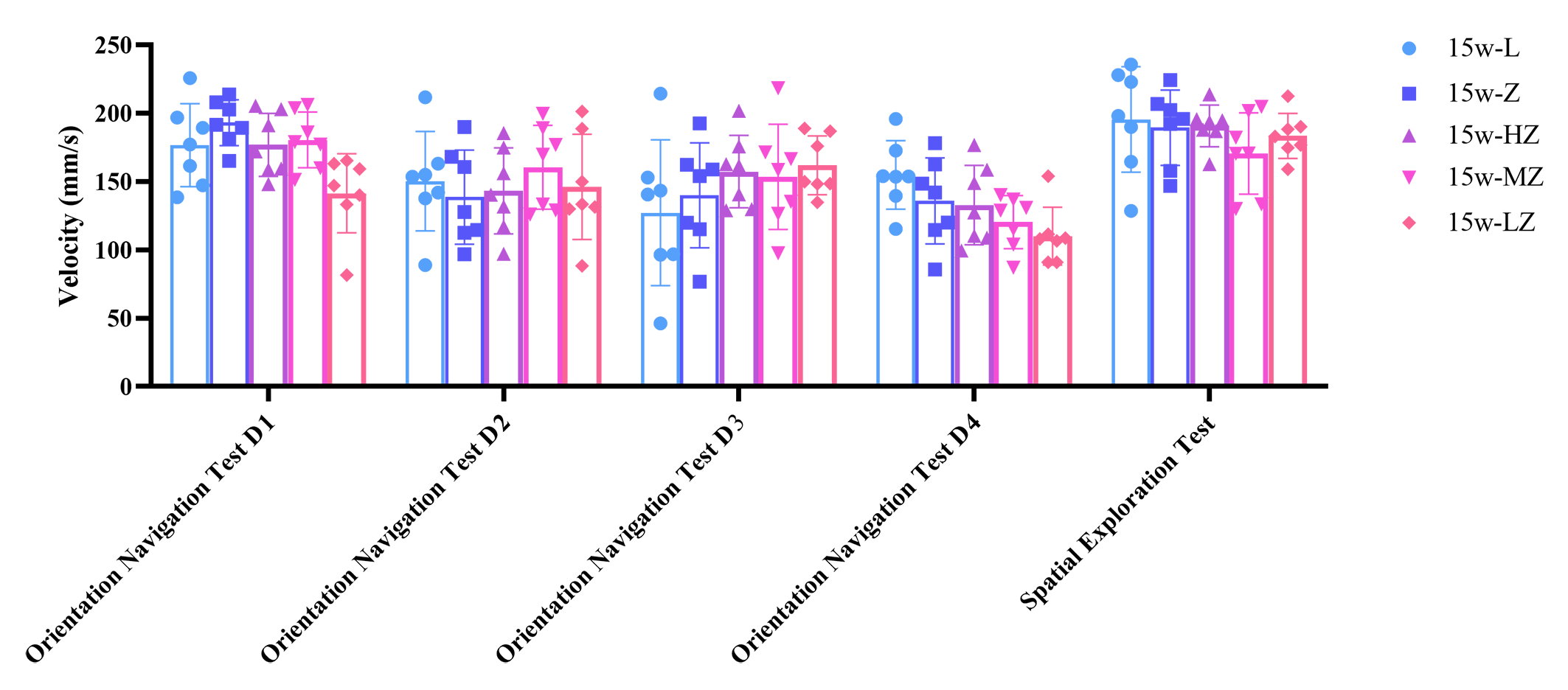

Supplement: Supplementary Figure 3 — Swimming speed in Morris water maze test of the orientation navigation test and the spatial exploration test. Data are shown as mean ± SD. n = 7 per group. D, day. [file Image_3.TIF]

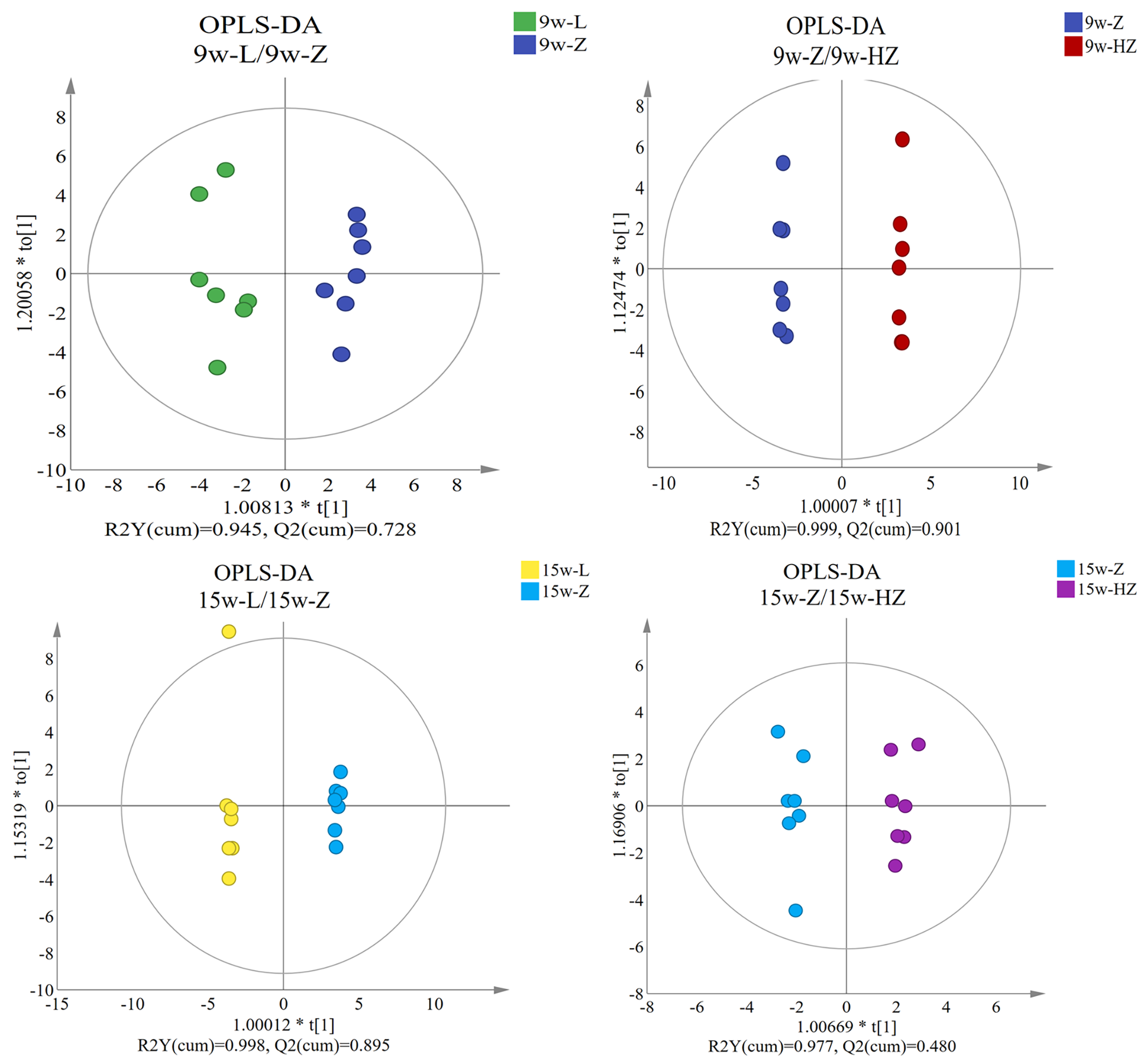

Supplement: Supplementary Figure 4 — OPLS-DA score plots for the different groups in the intestinal contents microbiology of rats. OPLS-DA, orthogonal partial least squares discriminant analysis. [file Image_4.TIF]

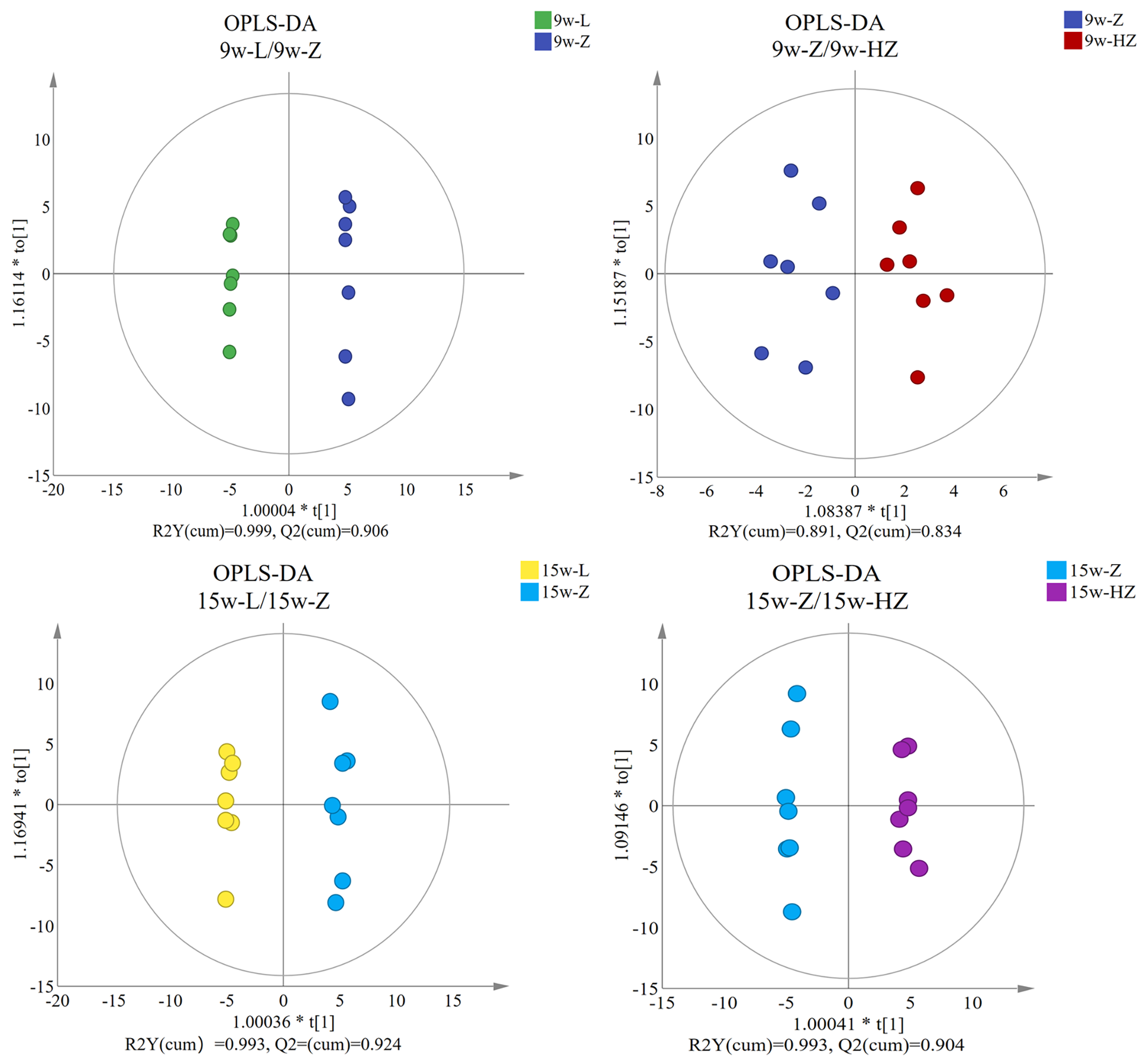

Supplement: Supplementary Figure 5 — OPLS-DA score plots for the different groups in the intestinal contents metabolomics of rats. OPLS-DA, orthogonal partial least squares discriminant analysis. [file Image_5.TIF]

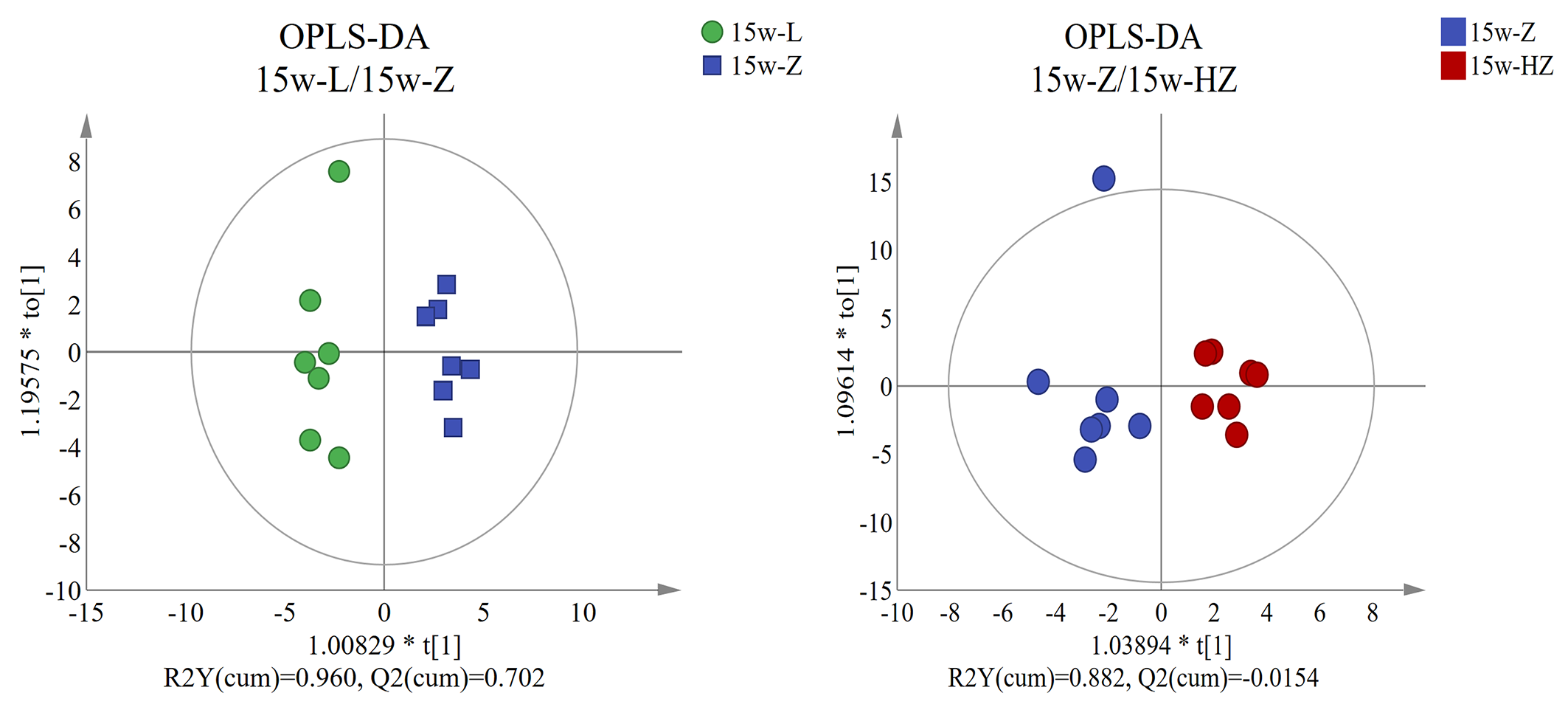

Supplement: Supplementary Figure 6 — OPLS-DA score plots for the different groups in the hippocampus metabolomics of rats. OPLS-DA, orthogonal partial least squares discriminant analysis. [file Image_6.TIF]
